# Cardamonin Attenuates Experimental Colitis and Associated Colorectal Cancer

**DOI:** 10.3390/biom11050661

**Published:** 2021-04-29

**Authors:** Shirley James, Jayasekharan S. Aparna, Anu Babu, Aswathy Mary Paul, Manendra Babu Lankadasari, Subha R. Athira, Sreesha S. Kumar, Yadu Vijayan, Narayanan N. Namitha, Sabira Mohammed, Girijadevi Reshmi, Kuzhuvelil B. Harikumar

**Affiliations:** 1 Cancer Research Program, Rajiv Gandhi Centre for Biotechnology (RGCB), Thiruvananthapuram 695014, India; shirlyjames@rgcb.res.in (S.J.); aparnajs90@gmail.com (J.S.A.); anub@rgcb.res.in (A.B.); aswathym@rgcb.res.in (A.M.P.); lamb.jan90@gmail.com (M.B.L.); athirasr@rgcb.res.in (S.R.A.); sreeshaskumar05@gmail.com (S.S.K.); yadu@rgcb.res.in (Y.V.); namithann@rgcb.res.in (N.N.N.); sabira.jazir@gmail.com (S.M.); reshmisuresh@gmail.com (G.R.); 2 Manipal Academy of Higher education (MAHE), Manipal 576104, India

**Keywords:** cardamonin, colitis, nutraceutical, colorectal cancer, azoxymethane, microRNA

## Abstract

Cardamonin is a naturally occurring chalcone, majorly from the Zingiberaceae family, which includes a wide range of spices from India. Herein, we investigated the anti-inflammatory property of cardamonin using different in vitro and in vivo systems. In RAW 264.7 cells, treatment with cardamonin showed a reduced nitrous oxide production without affecting the cell viability and decreased the expression of iNOS, TNF-α, and IL-6, and inhibited NF-kB signaling which emphasizes the role of cardamonin as an anti-inflammatory molecule. In a mouse model of dextran sodium sulfate (DSS)-induced colitis, cardamonin treatment protected the mice from colitis. Subsequently, we evaluated the therapeutic potential of this chalcone in a colitis-associated colon cancer model. We performed microRNA profiling in the different groups and observed that cardamonin modulates miRNA expression, thereby inhibiting tumor formation. Together, our findings indicate that cardamonin has the potential to be considered for future therapy against colorectal cancer.

## 1. Introduction

Colorectal cancer (CRC) is currently one of the most commonly diagnosed cancers worldwide. However, in several countries, CRC incidence rates in young adults (aged 40–50 years) are increasing, and the opposite trend is observed in older adults [1]. Much evidence has suggested that individuals with inflammatory bowel disease (IBD) are at an increased risk for developing CRC [2]. IBD consists of two types of idiopathic disorders (Crohn’s disease and ulcerative colitis) which lead to inflammation of the colonic mucosa. The cumulative risk of developing CRC is estimated to be as high as 30% for those with IBD [3,4].

Many epidemiological studies have clearly demonstrated a direct link between the usage of nutraceuticals and health benefits [5,6]. Nutraceuticals are dietary products with a cocktail of chemical components, including flavonoids, chalcones, etc., and show a therapeutic benefit on human health beyond their nutritional aspect and, in most cases, without any apparent side effects [7,8]. Cardamonin is a 2,4 dihydroxy-6-methoxy chalcone under the family of flavonoids. Cardamonin was reported to have several biological properties. This molecule enhanced phagocytic abilities of macrophages in WEHI-3-induced leukemic bearing mice [9]. In a breast cancer xenograft model, cardamonin decreased tumor growth and angiogenesis through HIF-1alpha inhibition [10]. This chalcone also inhibited the growth and induction of apoptosis in cancer cell lines of different origins [11,12,13,14,15]. Reports from our laboratory and others showed that cardamonin is a potent inhibitor of NF-kB, STAT3 [15,16], mTOR [17], and Wnt/β-catenin signaling [18]. Cardamonin also protects against cardiotoxicity and inflammation induced by chemotherapeutic drugs [19]. The involvement of chronic inflammation in the process of carcinogenesis is still an active area of research [20]. The alterations in microRNA expression have been shown to contribute to inflammation-driven colorectal cancer [21]. The Azoxymethane (AOM)-induced dextran sodium sulfate (DSS)-promoted colitis-associated cancer model (CAC) mimics the events that occur during human IBD and colon cancer [22]. In this study, we evaluated the protective effect of cardamonin in a colitis-associated cancer model (CAC) in mice. We observed that, through microRNA modulation, cardamonin exerts its anti-inflammatory and anti-cancer effects.

## 2. Materials and Methods

### 2.1. Reagents

Cardamonin (>98%) was procured from Shanghai Standard Biotech Co., Ltd., Shanghai, China. Azoxymethane was purchased from Sigma-Aldrich (St. Louis, MO, USA), dextrose sodium sulfate (DSS) was obtained from MP Biomedicals (Eschwege, Germany). All other reagents used in the study were of analytical grade.

### 2.2. Mice 

C57Bl/6 mice were originally purchased from Jackson Laboratories (Bar Harbor, ME, USA) and were housed in individually ventilated cages with standard rodent chow and water ad libitum and in a 12 h light/dark cycle. All animal experiments were performed with prior approval from the Institutional Animal Ethics Committee (IAEC) of RGCB and followed the guidelines of CPCSEA, Government of India (IAEC No 166).

### 2.3. Cell Lines and Cell Culture

HCT116, THP-1 and RAW 264.7 Cells were procured from ATCC (Manassas, VA, USA). HCT116 and THP-1 cells were cultured in DMEM and RPMI, respectively, with 10% fetal bovine serum. RAW 264.7 cells were grown in DMEM without phenol red, containing media with 10% fetal bovine serum and antibiotic-antimycotic solution. Cells were routinely checked for mycoplasma contamination using the MycoSensor PCR Assay Kit, from Agilent (Santa Clara, CA, USA).

### 2.4. Cytotoxicity by MTT Assay

RAW 264.7 cells (5 × 10^4^ cells/ well) were plated in 96-well plates and incubated overnight. The cells were treated with cardamonin at various dilutions and incubated for 24 and 48 h. Then, the media were removed and washed once with PBS. Subsequently, 100 µL of media containing 20 µL of MTT (5 mg/mL) was added to the cells and incubated for 4 h. Then, the medium was replaced with 100 µL of DMSO for solubilizing the blue crystals and the OD was taken at 490 nm with a reference of 655 nm in TECAN spectrophotometer.

### 2.5. NO measurement by Griess Reagent

RAW 264.7 cells (5 × 10^4^ cells/ well) were plated in DMEM medium without phenol red and incubated for 24 h. The cells were pre-treated with the required concentration of cardamonin and incubated for another 24 h. LPS treatment (100 ng/mL) was given 12 h. The media were collected and then proceeded for NO measurements using a Griess reagent kit. Briefly, 50 µL of the supernatant was collected and 25 µL of sulfanilic acid was added and incubated for 5 min. Then, 25 µL of NED reagent was added and incubated for 10 min and absorbance was read at 540 nm.

### 2.6. Real-Time PCR

The total RNA was extracted using TRIzol reagent and the RNA concentration was measured using nanodrop spectrophotometer. Then, 1.5 µg of RNA was converted to cDNA using an ABI high-capacity reverse transcriptase kit. The SYBR green-based real-time PCR was conducted using ABI step one plus real-time PCR system with gene-specific primers and GAPDH served as the housekeeping gene. The experiment was carried out in triplicates with three independent experiments and the fold change was calculated using the 2^−ΔΔCt^ method. The primer sequences used were as follows.

The primer sequences used as follows. i-NOS (Forward primer- AAACC CCTTGTGCTGTTCTCA; Reverse primer-GAACATTCTGTGCTGTCCCAGT), mIL-6 (Forward-CTGT AGCTCATTCTGCTCTGGA; Reverse-CAACTGGATGGAAGTCTCTT GC), mTNF (Forward-GTCCCCAAAGGGATGAGAAGTT; Reverse-ACAGGCTTGTC ACT CGAATTTTG), mSMAD2 (Forward-ATGTCGTCCATCTTGCCATTC; Reverse-AA CCGTC CTGTTTTCTTTAGCT), mOGT (Forward-TTCGGGAATCACCCTACTTCA, Reverse-TACCATCATCCGGGCTCAA), mRAB10 (Forward-CGATGCCTTCAAATA CCACCT; Reverse-GCCACTTGCTGATGTTCTCA), CUL4B (Forward-TATTAGTTGG CAAGAGTGCAT; Reverse-CCAGTAACCCATTGTCAGGAT), mSphK1 (Forward-CGT GGACCTCGAGAGTGAGAA; Reverse-AGGCTTGCTAGGCGAAAGAAG), mSphK2 (Forward- GCTTTCACCCATCGCTGAAG; Reverse GGCAGGAACCCCGAAGAT). 

### 2.7. Collection of Condition Media after Cardamonin Treatment

THP-1 cells were cultured, and cell supernatants were collected. The medium was centrifuged at 2000 rpm to remove any debris and then was added to HCT116 cells. After 24 h, an MTT assay was performed to check the cell proliferation experiment. In another experiment, THP-1 cells were treated with cardamonin (20 μM) for 24 h and were collected. For cell proliferation assays, cardamonin-treated conditioned medium was added to HCT116 cells, and cells were collected after 3 h (for RT-PCR analysis) and 24 h for MTT assay.

### 2.8. Immunofluorescence

Immunofluorescence was conducted to check the nuclear translocation of p65 that signifies the activation of the NF-κB pathway, the pro-inflammatory signaling cascade. RAW 264.7 cells (5 × 10^4^ cells/mL) cultured in polylysine-coated cover slips placed in 6-well plates were treated with 20 µM cardamonin for 24 h and treated with TNF-α 10 ng/mL for 30 min. The cells were fixed with 4% paraformaldehyde for 15 min followed by 1× PBS wash. The cells were then permeabilized with 0.1% Triton X for 10 min and washed with 1× PBS. Then, blocking was performed with 3% bovine serum albumin (BSA) (Invitrogen) for 30 min at room temperature and washed with 1× PBS. Primary antibody (p65–Santa Cruz Biotechnology 1:300 dilution) in 3% BSA was added to the smear and kept at 4 °C overnight in humidified chamber. The antibody was removed by washing with 1× PBS. Secondary antibody (Molecular Probes: Anti-rabbit Alexafluor 488, 1:400 dilution) in 3% BSA was added and incubated in a dark, humidified chamber for 1 h at room temperature and washed with 1× PBS, followed by counterstaining with 1 µg/mL Hoechst Bisbenzimide H 33342 (Sigma) for 10 min at room temperature. Finally, cells were washed with 1× PBS, mounted with DPX (Merck) and coverslip, and analyzed using an Olympus FV3000 confocal microscope with 60× magnification.

### 2.9. In Vivo Colitis Model and Cardamonin Treatment

Colitis was induced using dextran sodium sulphate (DSS). C57Bl/6 mice (6–8 weeks old, *n* = 5/group) were divided into the following groups: Group 1: Vehicle treated; Group 2: Cardamonin 10 mg/kg, p.o daily; Group 3: Cardamonin 50 mg/kg, p.o daily. The colitis cycle was performed as follows. The mice were treated with 5% DSS in drinking water for 6 days followed by drinking water for another six days. Mice were monitored daily, and body weights were taken. 

### 2.10. In Vivo Colitis-Associated Colorectal Cancer Model (CAC) and Cardamonin Treatment

The AOM and DSS induced colorectal cancer model has been described previously [23]. Briefly, 6–8-week-old mice (*n* = 10/group except for group 1 where *n* = 3) were divided into four groups: (a) Group 1: Mice without any treatment; (b) Group 2: Vehicle control, carboxymethyl cellulose was given during the entire experiment; (c) Group 3: cardamonin 10 mg/kg, administered orally, from day 1 to day 140; (d) Group 4: cardamonin 10 mg/kg, administered orally from day 50 (after completing the DSS cycles) to day 140. Groups 2, 3 and 4 were given AOM (10 mg/kg.b.wt., single dose, *i.p*) at day 0, and three bouts of 5% DSS at days 5–10, 25–30, 45–50. At day 140, the mice were sacrificed, and a gross necropsy was performed. 

### 2.11. RNA Isolation from Tumor Samples

A mirVana miRNA isolation kit (Thermofisher Scientific, Waltham, MA, USA) was used for total RNA isolation from the tumor samples as per the manufacturer’s protocol. Briefly, 20 milligrams of tissue were homogenized in the lysis buffer, and after that miRNA homogenate additive and acid:phenol:chloroform (APC) mixture was added to the lysed product and centrifuged. Then, the upper aqueous layer was collected, and RNA was precipitated using ethanol. Total RNA yield was determined using Nanodrop, and gel electrophoresis was used to confirm the integrity of isolated RNA samples.

### 2.12. MicroRNA Microarray of Tumor Samples and Analysis

GeneChip miRNA Array 4.0 (Affymetrix, Santa Clara, CA, USA) was used to profile the miRNAs and the entire procedure was undertaken based on the manufacturer’s instructions. Briefly, 1 µg of RNA was used for labelling with FlashTag Biotin HSR RNA Labeling Kit (Affymetrix), and hybridization was performed in a hybridization oven at 48 °C at a rotation of 60 per minute for 18 h. The miRNA chips were washed with GeneChip fluidic station 450 using an appropriate fluidic script and analyzed with a GeneChip command console. Microarray datasets obtained were analyzed as reported previously [16]. Significant mouse microRNAs were extracted from the list for further functional analysis. 

### 2.13. Computational Analysis for Target Prediction

We used three different target-predicting algorithms to predict the potential targets of the selected miRNA: miRDB with a cutoff target score less than 50, TargetScan with a total context score less than −0.36, and miRTarBase. 

### 2.14. Statistical Analysis

For microRNA analysis, Student’s unpaired *t*-test using R Bioconductor was used to compare the statistical difference between the groups. Significantly expressed miRNAs were identified based on a *p*-value ≤ 0.05. For other experiments, unpaired *t*-tests or ANOVA in GraphPad were used.

## 3. Results

### 3.1. Cardamonin Did Not Affect the Cell Viability but Inhibited Nitric Oxide (NO) Release in Lipopolysaccharide (LPS) Stimulated RAW 264.7 Cells

**Figure 1:**
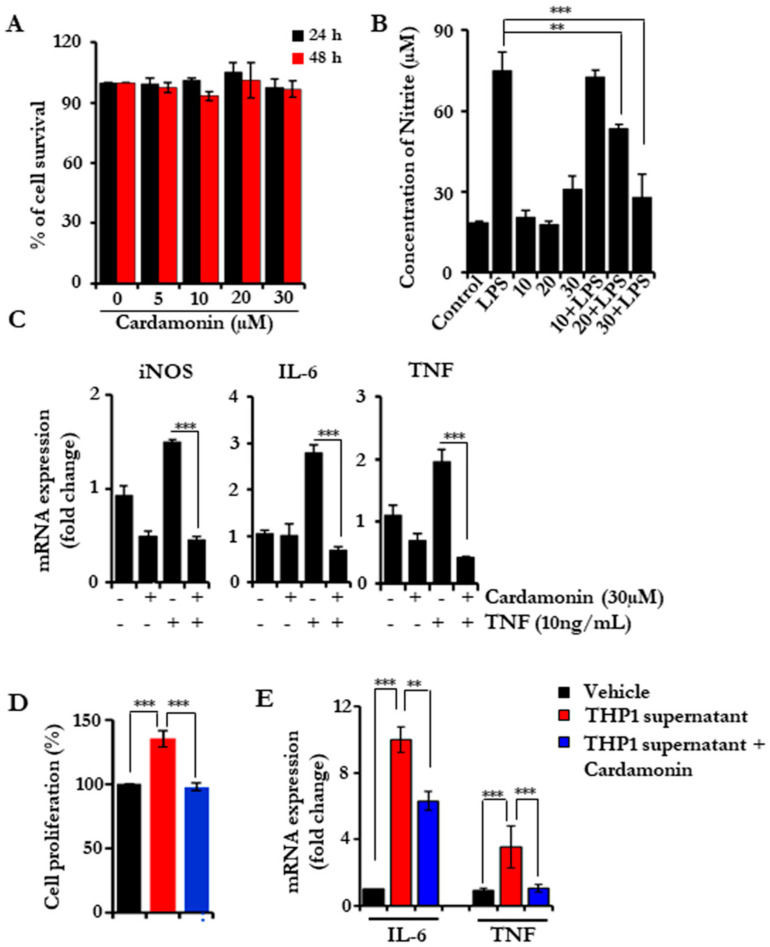
Cardamonin blocks the NO production in RAW 264.7 cells and reduces the inflammatory cytokine burden. (**A**) Cell proliferation assay in RAW 264.7 cells showing the cytotoxicity of cardamonin in a dose-dependent manner after 24 and 48 h of treatment. (**B**) Assessing the nitrous oxide levels released after LPS stimulation using Griess reagent after pretreating with cardamonin. (**C**) Quantitative PCR analysis for iNOS, IL6 and TNF-α levels in RAW 264.7 cells after treatment with cardamonin and TNF induction as indicated. GAPDH was used as the house-keeping gene. (**D**,**E**) HCT116 cells were cultured and THP-1 conditioned medium collected from THP-1 culture treated with either vehicle of cardamonin (20 μM; 24 h) was added. The cell proliferation was assessed after 24 h using an MTT assay (**D**); the RNA was isolated after 3 h and gene expression was analyzed using RT-PCR (**E**). Statistical significance was determined by Student’s *t*-test, ** *p* < 0.005, *** *p* < 0.001.

First, we sought to investigate the anti-inflammatory properties of cardamonin. For this, we chose RAW 264.7 cells, which is a well-established mouse macrophage cell line. Initially, we tested the cytotoxicity of cardamonin and found that it did not induce any cell death up to 30 µM concentration (Figure 1A). Next, we pre-treated the RAW 264.7 cells with 10, 20, and 30 µM concentrations of cardamonin and incubated them for 24 h followed by stimulation with LPS for the indicated time periods. LPS activate iNOS, which lead to the production NO and was analyzed by measuring the levels of nitrite [24]. The nitrite released into the cell culture supernatants was measured using Griess reagent [25]; reduced levels of nitrite were found, in a dose-dependent manner with the treatment of the drug, which is indirectly a measure for NO (Figure 1B). This showed that cardamonin could effectively block nitrous oxide production and release without affecting the proliferative potential of the cells.

### 3.2. Cardamonin Inhibited the Inflammatory Gene Expression

Next, we investigated the effect of cardamonin on the expression of various inflammatory genes. We pre-treated serum-starved RAW 264.7 cells with cardamonin for 24 h followed by TNF treatment for 6 h. Then, real-time PCR was performed to check the expression of TNF and IL-6. TNF induction leads to an increased expression of these genes, and cardamonin treatment effectively reduced the expression of both genes. Next, we checked the expression of iNOS, which is majorly responsible for NO production. The expression of iNOS was found to be reduced with cardamonin, which may be responsible for reduced NO production (Figure 1C). Next, we used the conditioned media (CM) from THP-1, which is considered a human monocytic cell line. HCT116 cells were exposed to CM for 24 h, and we observed that the cell proliferation rate increased in the presence of CM. We pre-treated THP-1 cells with cardamonin (20 µM) for 24 h, collected the CM, and assessed cell proliferation. The cardamonin pre-treated CM did not increase the cell proliferation. THP-1 CM is known to contain different pro-inflammatory cytokines and chemokines, and we hypothesized that these molecules might be involved in cellular proliferation. To test this, we treated HCT116 cells with THP-1CM with and without cardamonin, and checked the expression levels of IL-6 and TNF (Figure 1D,E). CM from vehicle-treated THP-1 induced the expression of both cytokines, while CM from cardamonin-treated cells inhibited the expression. These results further support the anti-inflammatory potential of cardamonin. 

### 3.3. Cardamonin Blocks the NF-κB Nuclear Translocation

**Figure 2:**
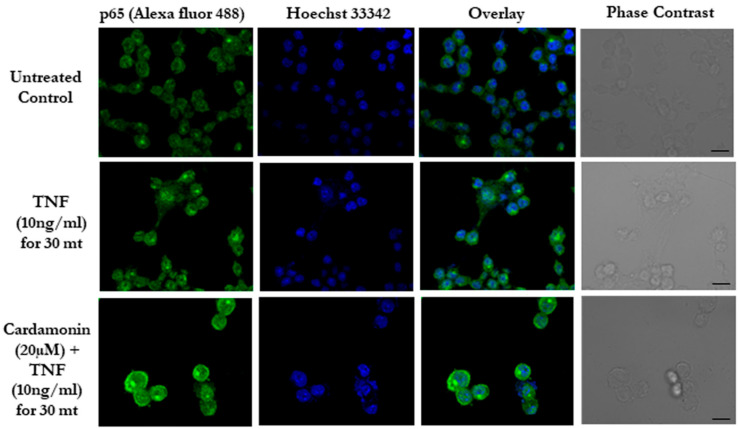
Cardamonin inhibits NF-κB signaling in vitro. Immunofluorescence of p65 (green) translocation in RAW 264.7 cells after induction with TNF along with pre-treatment with cardamonin. Hoechst (Blue) was used as a counterstain. Scale bar = 20 µm.

Most of the inflammatory genes were tightly regulated by certain transcription factors such as NF-κB [26]. p65 is one of the sub units of the NF-**κ**B complex with a DNA binding motif, and upon stimulation, the p65/p50 complex translocated to the nucleus and regulated gene expression [27]. Next, we checked whether cardamonin has any effect on the translocation of p65 into the nucleus. Our immunofluorescence results showed a TNF stimulation that led to the translocation of the p65 subunit to the nucleus and a reduced translocation of p65 in the cardamonin pre-treated samples. This indicates that cardamonin can block the movement of the NF-κB p65 subunit and thereby reduce the expression of inflammatory-related genes (Figure 2). These data correlate with our inflammatory gene expression data, where we found that the drug effectively reduced TNF-α and IL-6.

### 3.4. Cardamonin Attenuated the Colitis Elicited by DSS

**Figure 3:**
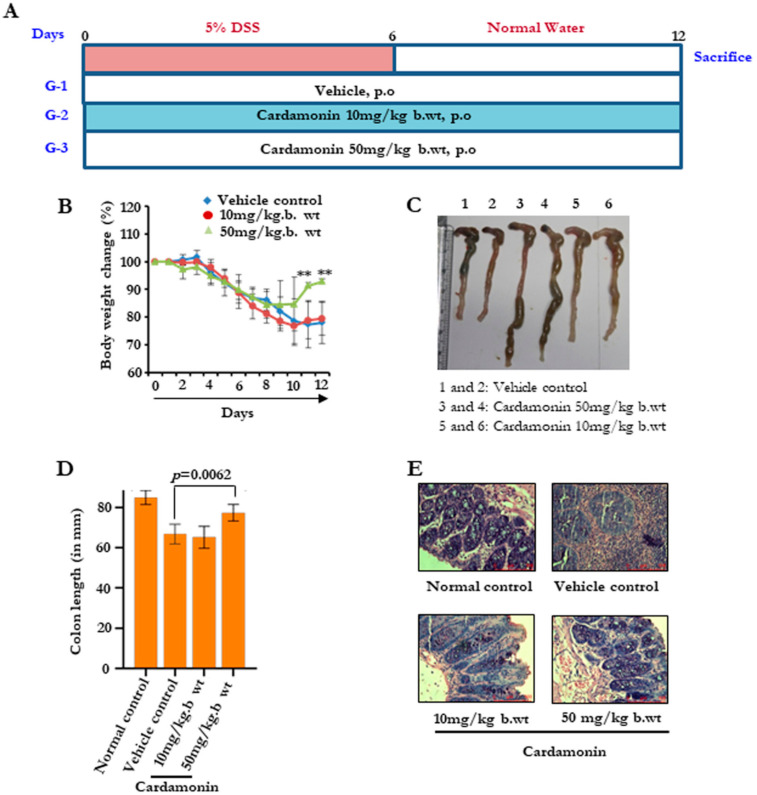
Cardamonin inhibits inflammatory signals and reduces DSS-induced colitis burden. (**A**) Schematic representation showing the time-bound drug treatments for establishing the DSS-induced colitis model. (**B**) The effect on body weight changes. Data are expressed as a percentage of body weight change. (**C**,**D**) The effect of cardamonin on colon length. (**C**) Representative pictures. 1 and 2, Vehicle control; 3 and 4, Cardamonin 50 mg/kg b.wt.; 5 and 6, Cardamonin 10 mg/kg b.wt. (**D**) Bar graph showing colon length in different groups on the last day of experiment. Statistical analysis was conducted using a *t*-test. (**E**) Representative H&E images of the colon from different groups. Scale bar = 100 µm.

Colitis is an important inflammatory condition that is associated with the defile villi, leading to injured pathology of the colon. Next, we investigated the effect of cardamonin, which we found to be an anti-inflammatory agent in vitro in the scenario of colitis. We developed a colitis model by supplying 5% DSS through the drinking water for six days, and for the remaining six days, normal water was supplied (Figure 3A). All the groups showed a drastic reduction in body weight from day 0 to day 6. During the recovery phase from day 7 to day 12, the body weight gain was much faster in the cardamonin 50 mg/kg group (Figure 3B). Then, after day 12, animals were sacrificed, a gross necropsy was performed, and the colon length was measured (Figure 3C,D). It was clearly evident that group 1, without any cardamonin treatment, showed an increased shrinkage in the colon, and this case was significantly reversed in the cardamonin-treated groups. We performed H&E staining to assess the changes in the pathology of the colon (Figure 3E). We found an accumulation of inflammatory cells in group 1, and a reduced accumulation in the rest of the groups. This showed that cardamonin reduces the inflammatory insult to the colon by blocking the accumulation of immune cells.

### 3.5. Cardamonin Reduced the Tumor Burden in the Colitis-Associated Colon Cancer

**Figure 4:**
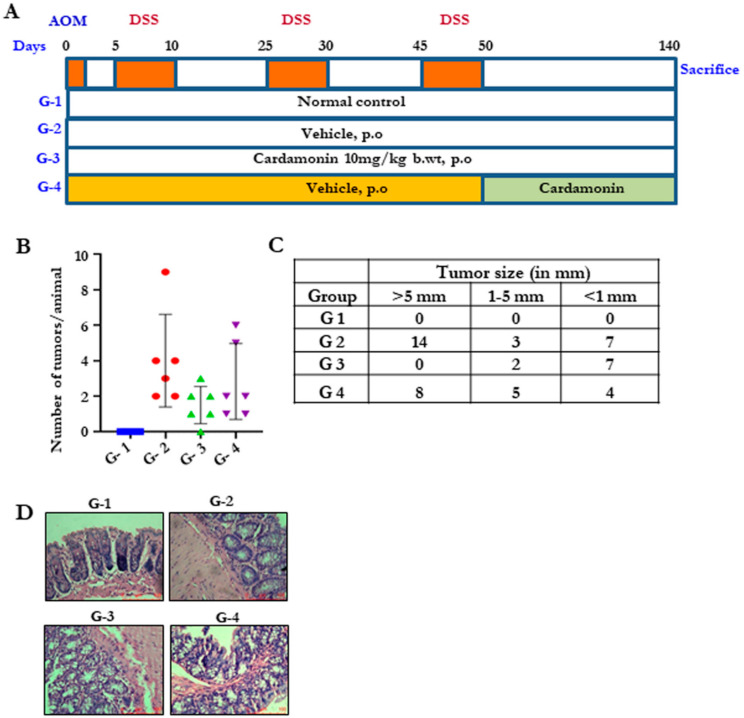
Cardamonin reduces tumor burden in colitis-associated colorectal cancer. (**A**) Schematic representation showing the time-bound drug treatments for establishing the AOM-induced, DSS-promoted, colitis-associated colorectal cancer model. (**B**) The effect of cardamonin on tumor number. The number of tumors per animal was counted and represented. (**C**) The effect of cardamonin on the size of the tumor (in mm). (**D**) H&E images of all four groups showing the tumor morphology, scale bar = 100 µm.

Colitis is one of the major risk factors leading to colon cancer. Therefore, next we determined whether cardamonin can reduce the risk of colitis-induced colon cancer. To do this, we developed an in vivo model by giving a single dose of AOM on day 0, followed by three bouts of 2.5% DSS for five days until day 50 (Figure 4A). Group 3 was administered with cardamonin five times per week, starting the next day after AOM injection (simultaneous model). In group 4, drug treatment began the next day after the last DSS cycle (developed model). We observed a significant reduction in the number of tumors in both group 3 and group 4 after necropsy. Animals were sacrificed at day 140. The number of tumors were counted in each animal, and we found that, on average, the number of tumors in the cardamonin-treated groups was reduced (Figure 4B,C). The tumors which appeared in cardamonin-treated groups were smaller in size as compared to vehicle-treated groups. Pathological analysis showed invasive tumors with the sensitized mucosal membrane, inflammatory cell infiltration, presence of dilated goblet cells and an undifferentiated epithelial morphology in group 2, and the above case was highly reversed in cardamonin-treated groups (Figure 4D), indicating that cardamonin protected the mice from colitis-associated colorectal cancer. 

### 3.6. Cardamonin Altered the miRNA Expression

**Figure 5:**
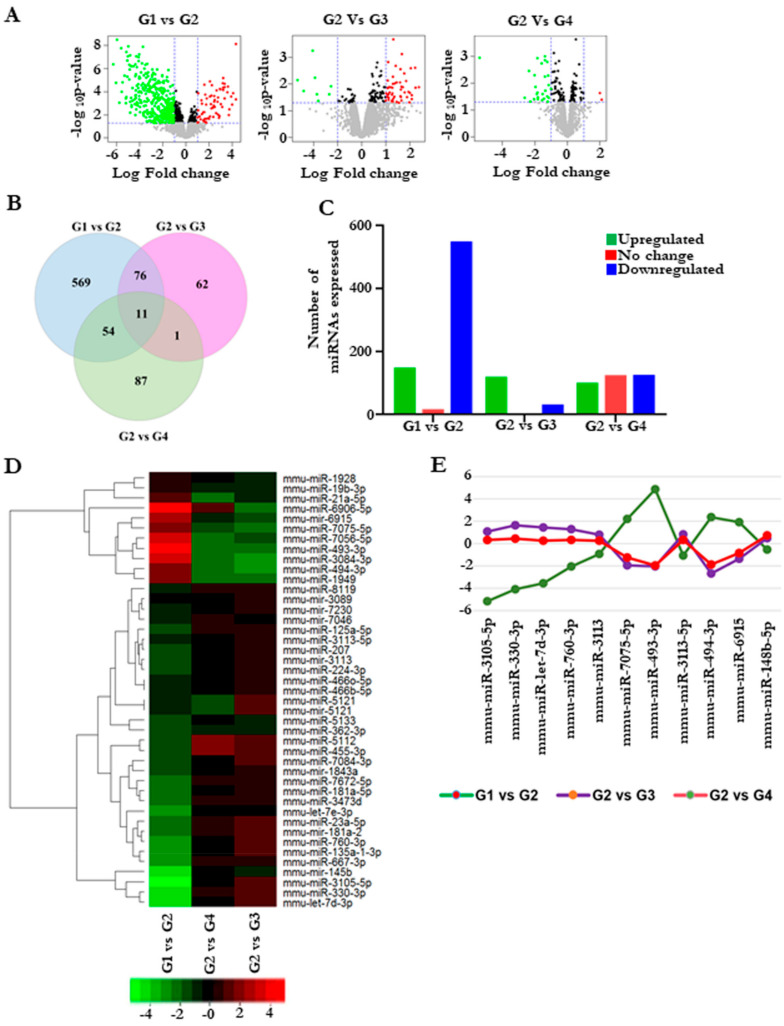
Cardamonin altered miRNA expression in colitis-induced colorectal cancer model. Group 1 (untreated normal control) was compared with group 2 (the vehicle control), and group 2 was further compared with groups 3 and 4 (the cardamonin-treated groups). (**A**) Volcano plot of the differentially expressed miRNAs from the microarray analysis of colon tumor samples. The abscissa represents the log2 transformation value of the differential expression as fold change. The *y*-axis represents the −log10 of the *p*-values. The *x*-axis represents the log fold change and is measured as the log2 transformed ratio of the expression between different experimental groups (G1 vs. G2; G2 vs. G3 and G2 vs. G4). Green dots represent significantly downregulated miRNAs, while red dots represent significantly upregulated miRNAs. (**B**) Venn diagram showing the total miRNAs overlapping between the groups. (**C**) The number of miRNAs expressed between different groups. (**D**) Heat map plot representing the differentially expressed miRNAs with the highest standard deviation (*p*  ≤ 0.10). The first column comprises G1VsG2, and the second and third columns comprise G2VsG4 and G2VsG3, respectively. (**E**) Graph showing selected miRNA expression intensity values (log scale) between the groups.

Next, we sought to understand the mechanism behind the protective effect of cardamonin. microRNAs (miRNAs) play an important role in regulating oncogenesis, and the modulation of them using drugs is of great interest. We investigated whether the reduction in the tumors was through alteration in the miRNA. To address this, we performed global miRNA microarray profiling to obtain the differentially regulated miRNA with and without cardamonin treatment. A volcano plot indicated that there was a differential expression of miRNAs between the groups (Figure 5A). A total of about 698 miRNAs were differentially expressed between group 1 and group 2, which clearly shows that miRNA modulation was one of the critical changes during colitis-associated cancer (Figure 5B,C). Figure 5Ds represent the heat map of miRNAs with significant expression with highest standard deviation (*p* ≤ 0.05). Six miRNAs were significantly downregulated and five were significantly upregulated in group 2 after cardamonin treatment (Figure 5E).

### 3.7. Validation of miRNA Microarray Data Using Their Targets

Next, we intended to validate the microarray results. This time, we followed the indirect validation by choosing a few targets of the miRNAs which showed significant fold changes between the groups. We selected SMAD2 [28], OGT [29], RAB10 [30] and CUL4B [31], which are targets for miR-494-3p, miR-330-3p, miR-3113-5p and miR-148b-5p, respectively. Here, miR-494-3p was upregulated during tumor induction and growth; therefore its target, SMAD2, should be downregulated. We found the trend followed by it according to its partner miRNA. RAB10 and CUL4B were upregulated in group 2, and expression levels decreased after the treatment. The OGT level was decreased only in group 3 and not in group 4 (Supplementary Figure S1). Thus, cardamonin regulates the expression of microRNAs and their targets in colitis-associated colorectal cancer. 

### 3.8. Developing an miRNA–mRNA Gene Interaction Network

**Figure 6:**
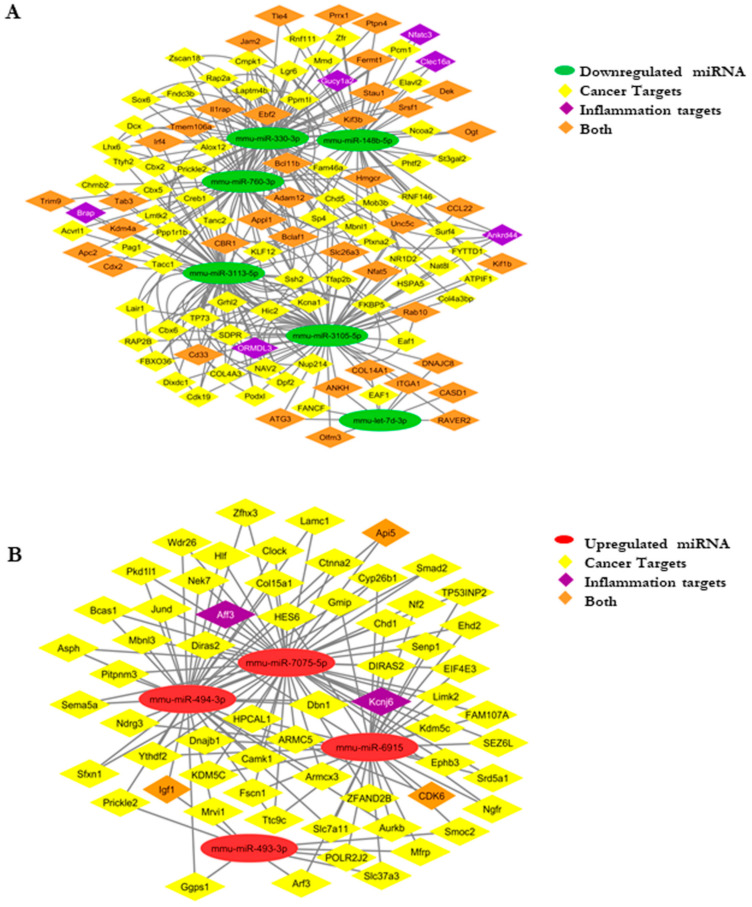
(**A**) miRNA–mRNA network: downregulated miRNA–gene network showing the targets specific for cancer, inflammation, and together. (**B**) The upregulated miRNA–mRNA network representing the targets specific for cancer, inflammation, and together.

Next, we decided to construct an interaction network using selected miRNAs with low *p*-values and their respective mRNA targets. We data-mined from targetscan (http://www.targetscan.org, accessed on 10 April 2017), dianatools (http://diana.imis.athena-innovation.gr/DianaTools/index.php, accessed on 10 April 2017), miRDB (http://mirdb.org/, accessed on 10 April 2017) and miRTarBase (https://bio.tools/mirtarbase, accessed on 10 April 2017) and selected the targets relevant in tumorigenesis. We selected the targets of the selected miRNAs to construct the interaction network. mmu-miR-6915, mmu-miR-493-3p, mmu-miR-494-3p, mmu-miR-7075-5p (upregulated in group 2), mmu-miR-3105-5p, mmu-miR-3113-5p, mmu-miR-330-3p, mmu-miR-760-3p and mmu-let-7d-3p, mmu-miR-148b-5p (downregulated in group 2). The upregulated and downregulated network is shown in Figure 6. The selection of the targets was performed from targetscan, with a cut off of less than −0.36. Then, the genes were manually sorted based on their functional roles and significance in cancer and inflammation, and the network (Figure 6A,B) was made using Cytoscape (http://www.cytoscape.org/, accessed on 12 January 2018).

### 3.9. Cardamonin Altered Crucial Pathways Involved in Cancer and Inflammation

**Figure 7:**
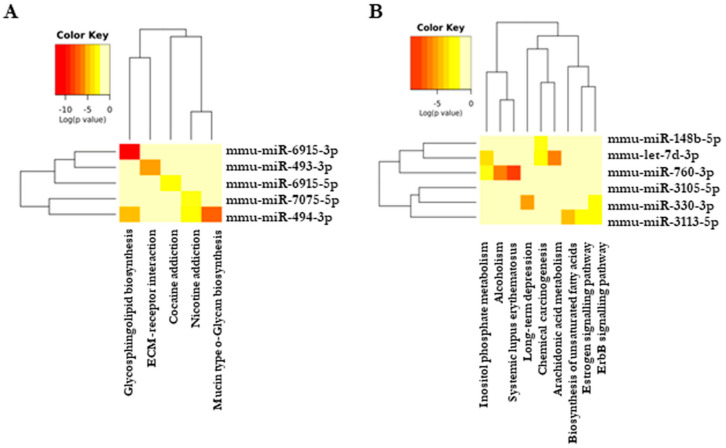
Cardamonin altered specific pathways important in cancer. (**A**,**B**) Dendrogram constructed using DIANA-miRPath v3.0 showing the pathways regulated by specific miRNAs; (**A**) upregulated; (**B**) downregulated. The darker colors represent statistical significance, as indicated in the color key.

To establish the functional correlation between the selected miRNAs and their regulated signaling pathways, we used DIANA-miRPath v3.0 (http://www.microrna.gr/miRPathv3, accessed on 8 August 2018) and prepared an miRNA cluster dendrogram which shows the common signaling pathways regulated by one or more miRNA using unsupervised hierarchical clustering. Our dendrogram result (Figure 7A,B) shows that most of the modulated miRNAs majorly regulate sphingolipid metabolism. Sphingolipid signaling plays a crucial role in inflammation and tumorigenesis [32]. To validate this observation, we checked the expression of sphingosine kinase 1 and 2 (SphK1 and 2) in all the four groups. There was significant upregulation of both kinase isoforms in vehicle-treated groups, and cardamonin significantly decreased the expression (Supplementary Figure S2). The other pathway, which was majorly regulated by more than two altered miRNAs, was ErbB signaling, which included prime molecules of carcinogenesis such as mTOR, MAPK’s and AKT. The AOM+DSS mouse model is a chemical carcinogenic model, and we also identified an miRNA, mmu-miR-330-3p, which regulates this process. In summary, cardamonin regulates the pathways involved in cancer and inflammation. 

### 3.10. Predicted Signaling Pathways Altered by miRNA Targets

**Figure 8:**
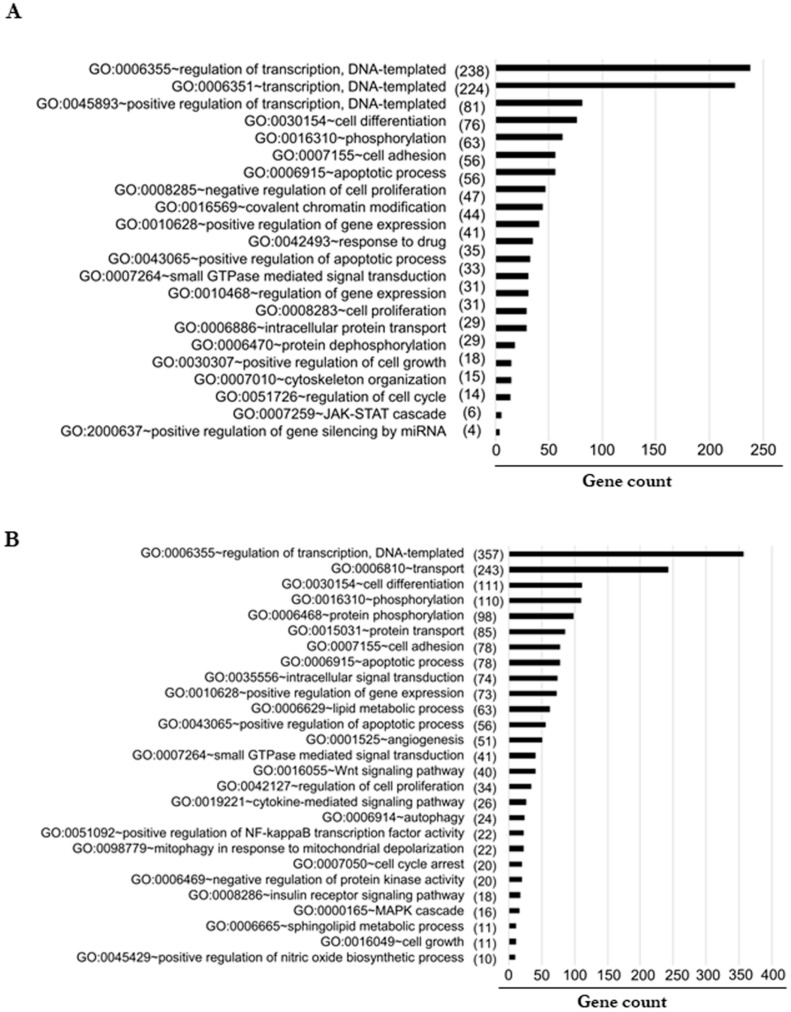
(**A**,**B**) Functional enrichment analysis using DAVID shows the significantly enriched biological process sorted in descending order based on the number of genes given in the parenthesis identified as targets for the downregulated (**A**) and upregulated (**B**) miRNA enriched in each process.

Next, we wanted to verify the major biological processes, which were altered during inflammation-induced tumorigenesis. To address this, we performed gene ontology enrichment analysis using DAVID Version 6.7 (http://david.abcc.ncifcrf.gov/, accessed on 12 January 2018). We selected all the miRNA gene targets from TargetScan with a cutoff of less than −0.36, and the selected gene targets were functionally annotated. We assessed the targets separately based on the altered miRNA. When we checked for their cellular localization, most of the altered proteins were localized to the nucleus, cytoplasm, and membranes. Then, we performed molecular functioning to check the major altered reactions in the cells. We found that the downregulated miRNAs majorly control phosphorylation, NF-κB transcription factor activity, MAPK cascade, lipid metabolic process, positive regulation of apoptotic process, and angiogenesis (Figure 8A). The upregulated miRNAs majorly control the function of proteins involved in the apoptotic process, negative regulation of cell proliferation, response to drugs, regulation of the cell cycle, and cytoskeleton organization (Figure 8B). These data strongly revealed that most of the proteins altered are majorly located in the nucleus and cytoplasm, which only regulate the prime functions of cells.

### 3.11. Cardamonin Altered miRNA Expression and Regulated the Crucial Molecules in the Cellular Pathways

**Figure 9:**
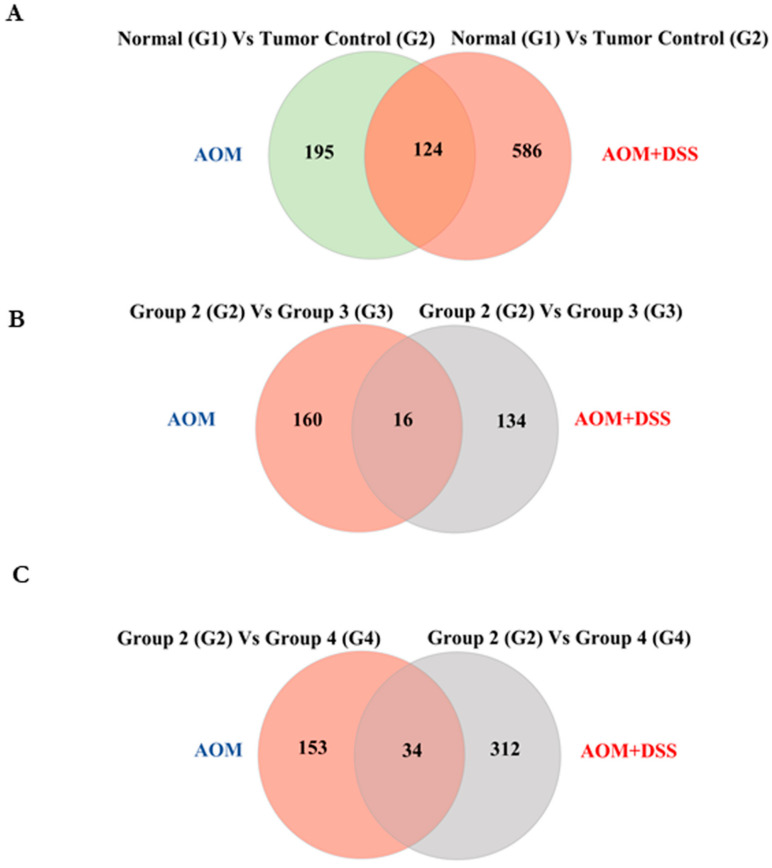
Comparison of miRNA expression in two models of colorectal cancer: azoxymethane (AOM)-induced colorectal cancer and AOM-induced, DSS-promoted colitis-associated cancer (AOM–DSS). Group 1 is untreated normal mice, group 2 is vehicle-treated group, group 3 is cardamonin treatment starting the next day after carcinogen injection (simultaneous), and group 4 is cardamonin treatment after tumor initiation. In the AOM model, drug treatment started from the 16th week after AOM injection, and in the AOM–DSS model, treatment started after the last DSS cycle. The Venn diagram of stably expressed miRNAs in the different conditions is depicted in (**A**–**C**).

We reported earlier the miRNA profile of azoxymethane (AOM)-induced colorectal cancer [16], and in this manuscript we have discussed the miRNA changes in the AOM–DSS model. These two are the most commonly used pre-clinical models of colorectal cancer. It was of great interest to compare both models and to see whether the expression of any miRNA was significantly altered in both models. As depicted in Figure 9A, there were 124 miRNAs whose expression was significantly altered in both models. To further evaluate the protective role of cardamonin, we assessed the miRNA expression of treatment groups in comparison with the vehicle control group (Figure 9B,C). We have listed the miRNAs whose expression was altered significantly in Supplementary Table S1. We observed that miRNAs dynamically regulated the carcinogenic process by modulating the cellular pathways, and cardamonin treatment regulated the process and inhibited colorectal cancer in both models.

## 4. Discussion

In this study, we provided evidence to support the anti-inflammatory activity of cardamonin. The compound inhibited NO production without affecting cell viability in LPS-stimulated mouse macrophages and the activation of TNF-driven NF-κB signaling. We used two doses of cardamonin for the colitis study, and the highest dose was more protective and prevented weight loss. In the DSS-promoted AOM-induced colorectal cancer model, both the treatment modalities showed significant therapeutic benefits. This is in agreement with another published study where continuous administration of cardamonin (40 mg/kg b.wt.) was found to reduce tumorigenesis [33]. In our study, we used a four-fold lower dose and observed the protective effect with two different treatment strategies. To delineate the mechanism of action, we conducted an analysis of the differential expression of miRNAs in different groups and observed a significant alteration in the global miRNA expression profiles between groups. Ren et al. also reported that cardamonin has the potential to inhibit Toll-like receptor pathways and MAPK signaling during colitis [34]. In a rat model of acetic acid ulcerative colitis, this chalcone inhibited the severity of colonic inflammation through downregulating TNF, iNOS and NF-κB signaling [35]. The resolution of inflammation is the most important therapeutic strategy in case of IBD-driven colorectal cancer. We have previously reported the therapeutic effect of cardamonin in AOM-induced colorectal cancer models and miRNA profiles [16]. Interestingly, we did observe several overlapping miRNAs in both models. Our studies imply that a set of distinctive miRNAs that are upregulated and downregulated during the initiation and progression of carcinogenesis and cardamonin dynamically regulate these microRNAs, suggesting the therapeutic potential of this spice-derived nutraceutical. 

Based on the previous report from our lab [16] and current study, we conclude that cardamonin: (a) is a potent anti-inflammatory agent and has the ability to suppress NF-κB and iNOS signaling; (b) protected the mice from inflammation-associated colitis; (c) modulated the expression of microRNAs; (d) inhibited the proliferation of colorectal cancer cell lines in vitro; and (e) increased the levels of reactive oxygen species and induced apoptosis. Cardamonin is a promising spice-derived nutraceutical with the potential to be developed as a future chemotherapeutic agent against colitis and colorectal cancer. 

## Data Availability

Microarray data are deposited at GEO with the accession number: GSE121608.

## Supplementary Materials

The following are available online at https://www.mdpi.com/article/10.3390/biom11050661/s1, Figure S1: Validation of selected gene targets: RNA was extracted from tumor samples and cDNA was prepared. The quantitative gene expression of SMAD2, OGT, RAB10 and CUL4b was performed using SYBR green approach. GAPDH was used as house keeping gene. Statistical significance was determined by Student’s *t*-test, *** *p* < 0.001, Figure S2: Cardamonin inhibited Sphingolipid metabolizing enzymes: RNA was extracted from tumor samples and cDNA was prepared. The quantitative gene expression of SphK1 and SphK2 was performed using SYBR green approach. GAPDH was used as house keeping gene. Statistical significance was determined by Student’s *t*-test, *** *p* < 0.001, Table S1: The list of miRNAs whose expression altered significantly between the groups in two mouse models of colorectal cancer.
