# Supplementary material for: Cardamonin Attenuates Experimental Colitis and Associated Colorectal Cancer
Source: Biomolecules. 2021 Apr 29;11(5):661. doi: 10.3390/biom11050661 (PMC8146383; doi:10.3390/biom11050661)
Supplement: Supplementary file 1 [file biomolecules-11-00661-s001.zip › biomolecules-1155711 - supplementary.pdf]

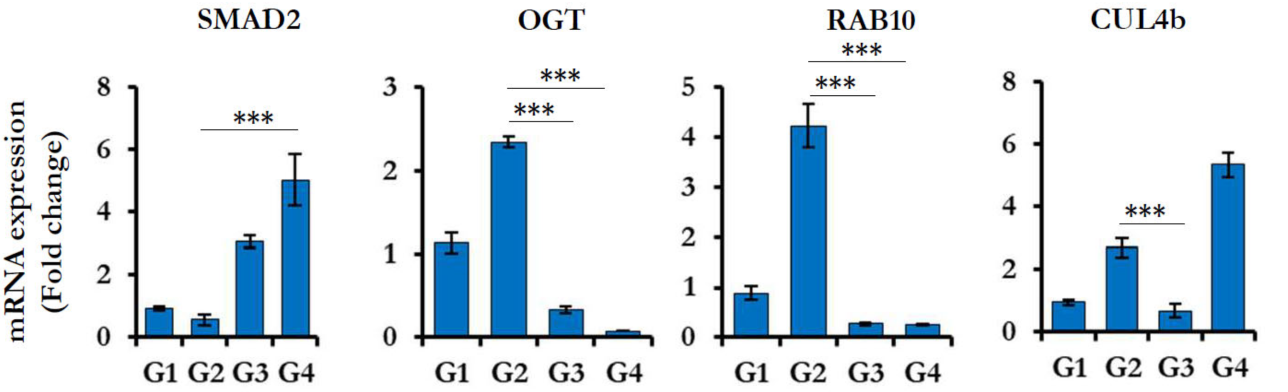


**Figure S1.** RNA was extracted from tumor samples and cDNA was prepared. The.quantitative gene expression of SMAD2, OGT, RAB10 and CUL4b was performed using SYBR greenapproach. GAPDH was used as house keeping gene. Statistical significance was determined by Student's *t*-test, *** $p < 0.001$.

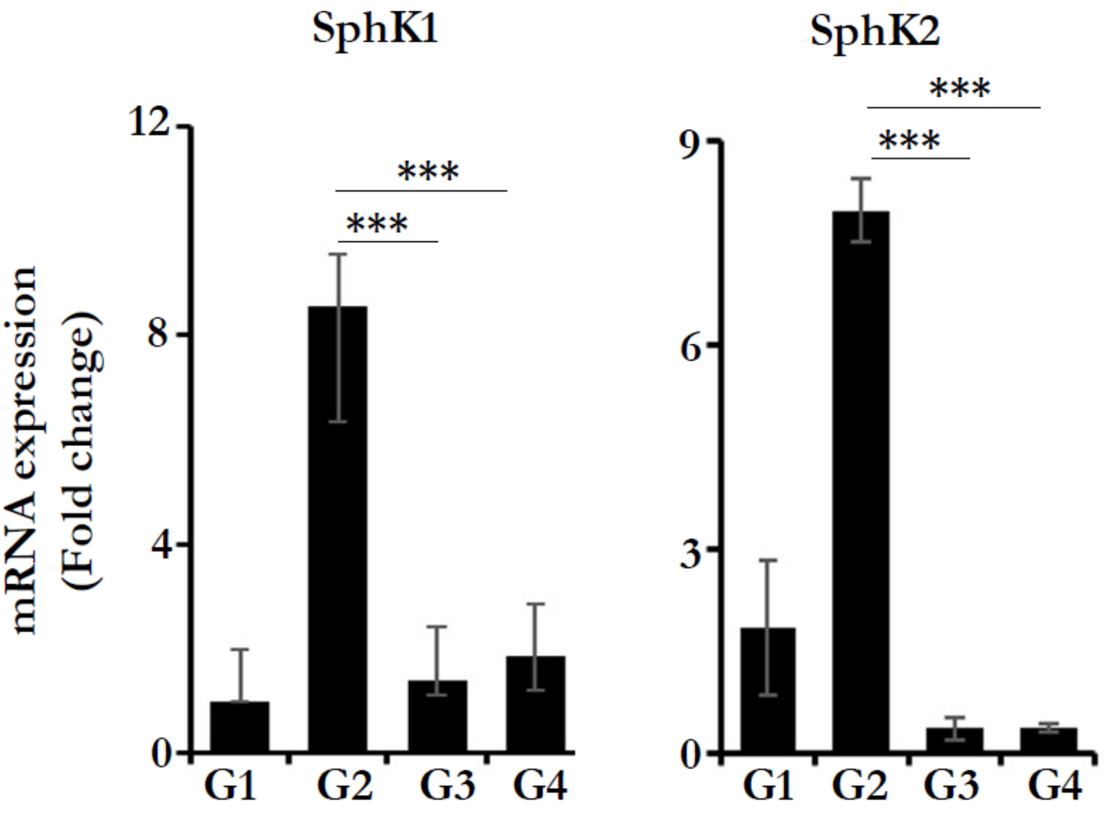


**Figure S2.** RNA was extracted from tumor samples and cDNA was prepared. The quantitative gene expression of SphK1 and SphK2 was performed using SYBR green approach. GAPDH was used as house keeping gene. Statistical significance was determined by Student's *t*-test, *** $p < 0.001$.
